# Medical specialty considerations by medical students early in their clinical experience

**DOI:** 10.1186/2045-4015-1-13

**Published:** 2012-03-12

**Authors:** Charles Weissman, Rachel Yaffa Zisk-Rony, Josh E Schroeder, Yoram G Weiss, Alex Avidan, Uriel Elchalal, Howard Tandeter

**Affiliations:** 1 Department of Anesthesiology and Critical Care Medicine, Hadassah-Hebrew University Medical Center, Hebrew University - Hadassah School of Medicine, Jerusalem, Israel; 2 Department of Nursing, Hadassah - Hebrew University Medical Center, Henrietta Szold Hebrew University - Hadassah School of Nursing, Jerusalem, Israel; 3 Department of Orthopedic Surgery, Hadassah-Hebrew University Medical Center, Hebrew University - Hadassah School of Medicine, Jerusalem, Israel; 4 Department of Obstetrics and Gynecology, Hadassah-Hebrew University Medical Center, Hebrew University - Hadassah School of Medicine, Jerusalem, Israel; 5 School of Continuing Medical Education and Senior Lecturer, Department of Family Medicine, Ben-Gurion University Joyce and Irving Goldman School of Medicine, Beer-Sheva, Israel

**Keywords:** Medical students, medical specialties, specialty selection, residency, healthcare system, physician workforce

## Abstract

**Background:**

Specialty selection by medical students determines the future composition of the physician workforce. Selection of career specialties begins in earnest during the clinical rotations with exposure to the clinical and intellectual environments of various specialties. Career specialty selection is followed by choosing a residency program. This is the period where insight into the decision process might help healthcare leaders ascertain whether, when, and how to intervene and attempt to influence students' decisions. The criteria students consider important in selecting a specialty and a residency program during the early phases of their clinical rotations were examined.

**Methods:**

Questionnaires distributed to fifth-year medical students at two Israeli medical schools.

**Results:**

229 of 275 (83%) questionnaires were returned. 80% of the students had considered specialties; 62% considered one specialty, 25% two, the remainder 3-5 specialties. Students took a long-range view; 55% considered working conditions after residency more important than those during residency, another 42% considered both equally important. More than two-thirds wanted an interesting and challenging bedside specialty affording control over lifestyle and providing a reasonable relationship between salary and lifestyle. Men were more interested in well-remunerated procedure-oriented specialties that allowed for private practice. Most students rated as important selecting a challenging and interesting residency program characterized by good relationships between staff members, with positive treatment by the institution, and that provided much teaching. More women wanted short residencies with few on-calls and limited hours. More men rated as important residencies affording much responsibility for making clinical decisions and providing research opportunities. More than 50% of the students considered it important that their residency be in a leading department, and in a large university medical center. Only 5% considered it important to do their residency in the country's peripheral areas, while 30% reported interest in a residency in the country's center.

**Conclusions:**

The fifth year of a six-year medical school is an opportune time to provide students with information and guidance on the various specialties and selecting a residency program as they begin to solidify their perceptions and ideas about the various specialties. This study serves as an impetus to medical educators and healthcare leaders to become interested in students' career selection.

## Introduction

Many students enter medical school with some idea of which medical specialty they wish to pursue [1-3]. However, the selection process begins in earnest during their clinical rotations when they are exposed to the clinical and intellectual environments of the various specialties. Choosing a career specialty is then followed by selecting a residency program. Much of the research performed on the medical specialty selection process has generally focused on students before or immediately upon beginning medical school, on students in their final year of medical school, or interns. A unique aspect of this study is that relatively few studies, and no Israeli ones, have examined the early clinical experience, defined as the fourth and fifth years in six-year programs and the third year in four-year programs. The early clinical experience is the first time most students have experienced the clinical arena. It is a period when initial impressions are made about the practical aspects of medical practice and also about the various specialties. Therefore, gaining insight into students' criteria for selecting medical specialties and residency programs during this period might provide an understanding of the nature of the early decision process. This information should be important for healthcare system leaders since specialty selection determines the future composition of the physician workforce. This improved understanding of the selection processes should also help these leaders ascertain whether, when, and how to intervene in an attempt to influence these decisions. The early clinical experience is likely an opportune time to begin such interventions because upon starting medical school up to 82% of students indicate interest in a specific specialty, but only 20-45% ultimately choose a residency in that specialty [4]. Others have shown that up to 80% change their specialty preferences from the time they take their medical school entrance examination until their final year of medical school [5].

Influencing students' choice of medical specialties may be especially important in Israel where there is a maldistribution of physicians among the various specialties. Notably, specialties such as ophthalmology and plastic surgery enjoy much popularity, while anesthesiology and general surgery are suffering existential shortages [6,7]. Residency program selection is also important since it can determine the geographic distribution of the physician workforce. In many instances the location of the residency program is a major determinant of where a young physician will decide to practice [8].

Under the Israeli six-year medical school curriculum, students begin their clinical experience in the fourth year. This initial experience involves rotations in internal medicine, pediatrics, and, in some schools, general surgery, while the fifth year includes rotations in additional specialties, such as psychiatry and obstetrics/gynecology. We performed a study exploring various aspects of the selection process among fifth-year students. Our aim was to investigate the criteria students consider important in their choices of both a specialty and a residency program at this point in their clinical experience. These data were used to determine whether the fifth year of medical school was an appropriate time to begin counseling students as to their career specialty selection.

## Methods

We designed a questionnaire to examine various aspects of the medical specialty selection process. This was performed by examining the contemporary literature about specialty and residency program selection. The questionnaire included multiple choice questions, free-text questions, and 5-point Likert scales. In addition to demographic information, the questionnaire elicited information on (1) reasons for choosing a medical career {12 items}; (2) whether they had already considered a specialty for their residency and, if so, which specialty (free text); (3) criteria for choosing a career specialty {15 items}; (4) criteria for choosing a residency program {17 items}; and (5) considerations on where to undergo residency training {9 geographic and hospital-related characteristics}.

After two small (15 students) preliminary studies designed to identify problems and test the user-friendliness of the questionnaire, the questionnaires were distributed to the fifth-year classes of the Hebrew University - Hadassah School of Medicine in Jerusalem during the 2008-2009 and 2009-2010 school years, and the 2009-2010 school year of the Ben-Gurion University Joyce and Irving Goldman School of Medicine in Beer-Sheva.

An *a priori *decision was made to separately analyze and compare the data from male and female students. This decision was based on prior research demonstrating significant gender differences associated with specialty selection [9]. The responses to multiple choice questions are presented as frequency distributions. For presentation, the two positive and two negative points of the 5-point Likert scales were combined and positive, neutral, or negative points reported. For statistical analysis all 5 points were used.

The reasons for choosing a medical career, criteria for specialty selection, and criteria for deciding on a residency program were subject to factor analysis using varimax rotation with factors having eigenvalues of ≥ 1.0. For continuous data differences between the male and female data, analysis used paired 2-tailed t-tests. Categorical data were analyzed using χ^2 ^tests. Cohen's κ statistic was used to examine the concordance between similar questions within the questionnaire. Logistic regression analysis was used to determine the characteristics of the students who had not identified a specialty for residency. Data analysis was performed using Microsoft Excel (Redmont, WA) and Systat 12 (San Jose, CA).

The Institutional Review Board of the Hadassah Medical Organization approved this study.

## Results

**Figure 1:**
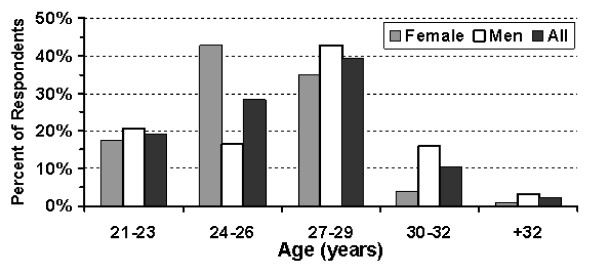
**The age distributions of the students are shown**. Male medical students were older since in Israel men generally serve in.

**Figure 2:**
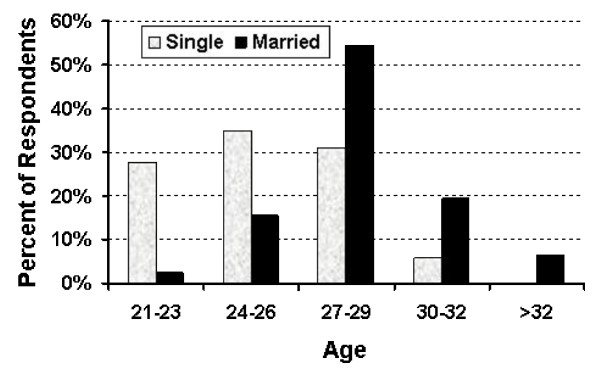
**The age distributions of married versus single students are shown**. Married medical students were older. All those over the age of 32 years were married.

Two hundred twenty-nine of 275 (83%) questionnaires were returned (175 of 200 from the Hebrew University - Hadassah School of Medicine; 54 of 75 from the Ben-Gurion University Joyce and Irving Goldman School of Medicine). There were no significant differences between the replies from the students of the two medical schools. One hundred four of the respondents (45%) were female. The age distributions are shown in Figure 1a. One hundred fifty-one students (66%) were single, 76 (33%) were married (35 had children), and the remaining 2 were widowed or divorced. The married students were older (Figure 2). Twenty-eight percent of the women and 37% of the men were married. Twenty-three of the men and 12 of the women had children. The reasons that the students chose to study medicine are shown in Table 1.

**Table 1:** Reasons for Choosing a Medical Career.

Question (factor)^‡^	All	Women	Men
	(n = 229)	(n = 104)	(n = 125)
Helping patients	74%	71%	78%
The scientific basis of medicine (2)	36%	40%	36%
Employment security	27%	23%	31%
The profession's image	21%	13%	27%*
Economic potential (1)	16%	11%	19%*
Potential for family life (1)	14%	15%	13%
Influence of a role model			
(family/friends) (3)	13%	11%	15%
Influence of previous studies	12%	14%	11%
Opportunity to perform research (2)	12%	10%	14%§
Influence of a role model			
(physician) (3)	12%	11%	13%
Illness of relative/friend	10%	17%	4%§
Previous employment experience	4%	5%	3%

Values are important/very important answers on a 5 point Likert Scale

vs. women: * *p *< 0.001, § *p *< 0.03

**^‡ ^**Factors are the results of the factor analysis performed on all the data

Eighty percent of the students (85% of the women and 75% of the men, *p *< 0.03) had already considered specialties for their residency. Sixty-two percent reported considering one specialty, 25% reported two, 11% reported three, and the remainder 4-5 specialties. Details of the specialties are found in Table 2. There was no age or marital status difference between those who had and had not considered a specialty. However, gender was a factor, with a significantly greater percentage (*p *< 0.04) of those who had not considered a specialty being male (66%). Among the students who had considered a specialty only 52% were male. This was also reflected by the results of logistic regression where female gender was significantly associated with having considered a specialty (odds ratio 2.64 [95% CI: 1.042-6.391] *p *< 0.031).

**Table 2:** The Medical Students' Specialty Selection.

Specialties	WOMEN (n = 86)		MEN (n = 90)	
**Pediatrics**	36	42%	27	30%
**Internal Medicine**	21	24%	20	22%
**Gynecology**	19	22%	9	10%
**Psychiatry**	11	13%	5	6%
**Neurology**	6	7%	7	8%
**Family Medicine**	4	5%	4	4%
**Ophthalmology**		5%	3	3%
**Oncology**	4	5%	0	0%
**Otolaryngology**	3	3%	3	3%
**Cardiology**	2	2%	4	4%
**Orthopedic Surgery**	2	2%	7	8%

**Figure 3:**
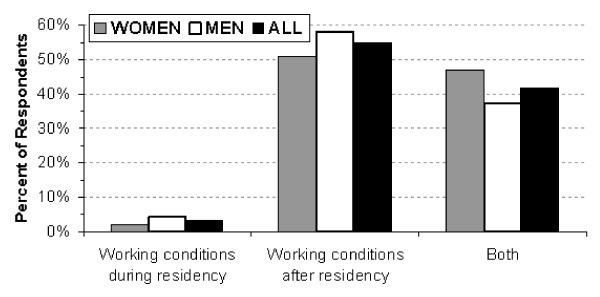
**Shown are the responses to the query: Which is more important when choosing a specialty, working conditions after residency, working conditions during residency, or both?**.

**Figure 4:**
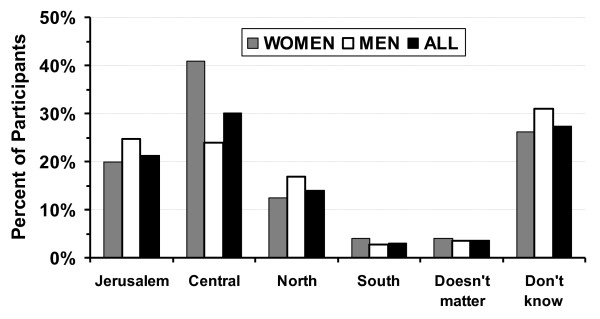
**The locations where the students would like to do their residency training**. The Hebrew University - Hadassah School of Medicine is located in Jerusalem, while the Ben-Gurion University Joyce and Irving Goldman School of Medicine is in the Southern Region. The Central Region is the major population center (Tel Aviv and its environs).

The criteria the students reported as being important/very important when considering a specialty as a career are revealed in Table 3 while Table 4 reports the criteria considered important/very important when choosing a residency program. Logistic regression revealed that the married students were less likely to seek a specialty with time in the operating room (odds ratio 0.56 [95% CI: 0.37-0.84] *p *< 0.005) and more likely to want a controllable lifestyle (odds ratio 1.54 [95% CI: 1.0-2.38] *p *< 0.05]. The analysis also showed that they were less likely to consider a residency in a specialty with much action (odds ratio 0.613 [95% CI: 0.44-0.84] *p *< 0.004) or a leading department in the specialty (odds ratio 0.479 [95% CI: 0.30-0.76] *p *< 0.002), but more likely to consider a residency with much responsibility (odds ratio 2.06 [95% CI: 1.301-3.28], *p *< 0.002). In addition, when the students were asked which was more important when choosing a specialty, 55% answered working conditions after residency, 3% answered working conditions during residency, and 42% reported that both were equally important (Figure 3). The follow-up question showed that when deciding on a residency, the specialty (77%) was more important than the department (1%). The remaining students reported that both were equally important. Criteria for selecting the location of a residency are found in Table 5 and preferred locations are found in Figure 4.

**Table 3:** Which of the Following will have a Positive Influence on your Selection of a Specialty as a Career?

Question (Factor)^‡^	ALL	WOMEN	MEN
	(n = 229)	(n = 104)	(n = 125)
Interesting/challenging specialty (2)^‡^	91.3%	90.0%	92.4%
A reasonable relationship between salary/lifestyle (1)	72.6%	72.0%	73.1%
Bedside specialty	67.9%	72.7%	63.9%
Control over lifestyle (1)	67.0%	73.7%	61.3%
A specialty that is rapidly advancing (2)	56.9%	53.5%	59.7%
Independent practice (1)	54.1%	52.0%	55.9%
High paying specialty (1)	49.5%	41.4%	56.3%*
Opportunity for private practice (1)	47.5%	41.0%	52.9%*
Performing surgery/procedures (3)	46.1%	35.0%	55.5%*
Much teamwork (2)	36.7%	36.0%	37.3%
Time in the operating room (3)	32.6%	22.0%	41.5%*
Opportunity to perform research(2)	29.8%	28.0%	32.5%§
Work only during the daytime	28.4%	40.0%	18.6%*
Work only in the hospital	13.8%	14.3%	13.4%
Specialties classmates choose (4)	0.5%	0.0%	0.9%*

Values are important/very important answers on a 5 point Likert Scale

vs. women: * *p *< 0.005, § *p *< 0.04

‡ Factors are the result of the factor analysis performed on all the data

**Table 4:** Which of the Following will have a Positive Influence on your Selection of a Residency Program?

Question (Factor)‡	ALL	WOMEN	MEN
	(n = 229)	(n = 104)	(n = 125)
Good relationships with attendings (3)‡	91.2%	93.9%	89.1%
Good relationships between residents (3)	89.0%	90.9%	87.4%
Interesting and challenging residency	86.8%	82.0%	90.8%
Residency with much resident teaching (3)	82.9%	85.9%	80.5%
Leading department in the specialty (4)	72.6%	72.0%	73.1%
Control over lifestyle (1)	63.9%	71.0%	58.0%§
A specific location within Israel	60.7%	66.0%	56.3%
A large university hospital (4)	57.1%	54.0%	59.7%
Much responsibility/Make clinical decisions on their own (2)	51.6%	45.0%	57.1%§
Working hours known from the start (1)	49.1%	57.6%	42.0%§
A residency with much "action" (2)	42.9%	36.0%	48.7%§
Residency with limited hours (1)	31.5%	39.0%	25.2%*
A residency with few on-calls (1)	29.2%	37.0%	22.7%*
Opportunity to perform research	23.3%	20.0%	26.1%§
Short residency (< 4.5 years) (1)	20.6%	26.0%	16.1%*
Work under pressure (2)	17.4%	16.0%	18.5%
Hospital in the country's periphery (4**)	4.6%	6.1%	3.4%

Values are important/very important answers on a 5 point Likert Scale

vs. women: * *p *< 0.005, § *p *< 0.04

‡ Factors are the result of the factor analysis performed on all the data

** a reciprocal (negative) member of factor #4

**Table 5:** Considerations when Choosing a Location for Residency.

	ALL	WOMEN	MEN
	(n = 229)	(n = 104)	(n = 125)
Excellence of medical center	88.7%	75.8%	96.2%*
General quality of life	71.3%	75.8%	64.8%*
Positive treatment by the institution	71.3%	73.7%	65.7%*
Employment location of significant other	69.2%	66.3%	68.6%
Availability of a residency position	59.0%	54.7%	60.0%
Close to family: parents/significant other's parents	49.2%	47.4%	48.6%
Close to the county's center	31.8%	32.6%	29.5%
Location of children's/significant other's studies	27.7%	31.6%	31.4%
Location of medical school	10.3%	10.5%	9.5%

vs. women: * *p *< 0.005

More female than male students indicated interest in pursuing careers in pediatrics, internal medicine, and family medicine (Table 2). Furthermore, students interested in these specialties were more often married than those interested in other specialties (41% vs. 29%, *p *< 0.03). Both women and men interested in these specialties were less interested (*p *< 0.03) in spending time in the operating room performing surgery/procedures or in a high paying specialty. They were also more likely to be interested in a residency in the peripheral (more rural) areas of the country (*p *< 0.05). In addition, women students interested in primary care were less interested than the other women in having a controllable lifestyle (*p *< 0.005) or pursuing an interesting and challenging specialty (*p *< 0.02). Male students interested in these specialties were less interested than other male students in a rapidly advancing specialty (*p *< 0.01), having a private practice (*p *< 0.001), or a residency with much "action" (*p *< 0.001).

The concordance of the responses was examined by comparing answers concerning interest in performing research in the different sections of the questionnaire: Reasons for choosing a medical career (Table 1) - opportunity to perform research vs. positive influence on specialty selection; (Table 2) - performing research: (Cohen's κ = 0.78); and positive influence on residency selection (Table 3): (Cohen's κ = 0.83). The relationship between the latter two answers was κ = 0.90. Cohen's κ was 0.77 between whether the students were looking for a career specialty with a controllable lifestyle or whether their residency program should afford a controllable lifestyle.

## Discussion

Already in their fifth year of medical school, Israeli students displayed definite opinions about criteria they would use for selecting residencies and specialties. Furthermore, some of the specialty and residency program selection criteria deemed important/very important by the Israeli students, such as the interest among both male and female students in choosing specialties and residency programs affording controllable lifestyles, have also been rated as important criteria by students in other countries [10]. In addition, 80% of the fifth year students had thought of career specialties, although over a third of them were considering more than one specialty. Whether these fifth-year interests translate into final choices needs to be determined, since some studies have shown that interests and attitudes expressed during the initial years of medical school are not necessarily translated into final choices [11]. In Israel a rotating internship year is required to obtain an MD degree, so that final specialty and residency program selections are usually only consummated during that year. Even though interests in specific specialties have been found in other studies to continually change during medical school and internship, this study shows that distinctive patterns, e.g., those of male vs. female students, of opinions, biases, and criteria, are already evident during the fifth year of medical school [4]. These findings should be of interest to medical educators and leaders of the healthcare system since they show that interventions, such as career counseling, aimed at assisting and possibly influencing the medical students' specialty choices should begin during the early phases of the clinical experience. This counseling should be aimed at the individual, rather than the group, since the students almost unanimously answered that they would not be influenced by the choices of their peers (Table 3).

Medical students are faced with a two-stage decision process. Initially, they must choose a medical discipline as a career and then select a residency program for the requisite training. Both stages of this process were examined in this study and it was found that students tended to take a long-range view, with 55% considering the working conditions after residency to be more important than those during residency, and another 42% considering both to be equally important. Moreover, the students overwhelmingly indicated that it was the specialty (77%) rather than the department (1%) that was more important when deciding on a residency program. These observations strengthen the arguments that when recruiting students to a specialty much of the thrust should be on the challenges, rewards, working conditions, and remuneration after residency. This does not preclude providing information about a specialty's residency requirements or a department's training program, but it is important to realize that this information is often secondary, since the students' initial major decision is which medical discipline to pursue.

This study also examined the importance assigned by students to various criteria used to select a specialty and then a residency program. More than two-thirds of the men and women indicated that they wanted an interesting and challenging bedside specialty that afforded control over their lifestyle and provided a reasonable relationship between salary and lifestyle. This importance given by both men and women medical students to controllable lifestyle and the relationship between remuneration and lifestyle has also been observed among medical students in countries such as the United States and Canada [12]. It has been ascribed to the desire of this generation to lead lifestyles that balance work and family/leisure activities [13]. The latter was further reflected in the female, as opposed to the male, medical students wanting to pursue specialties that allow for daytime work only. In other studies this criterion was associated with the emphasis placed by women students on family life and raising children [14]. This was further shown by the interest the women students displayed in pediatrics and psychiatry, which in Israel are specialties with controllable lifestyles. Alternately, the men were more interested in specialties that were procedure/surgery oriented and were well remunerated, ostensibly by providing opportunities for private practice. This pattern was also demonstrated by more men than women replying that they had chosen medicine because of its economic potential. Comparable gender-associated selection criteria patterns have been reported from other parts of the world [15]. However, with the proportion of female medical students frequently exceeding 50% in many countries, the surgical specialties are beginning to adapt and become more attractive to them [16,17].

Criteria for choosing a residency program and its location yielded significant differences between male and female students that mirrored the responses to the queries about specialty selection. Namely, significantly more women wanted short residencies with few on-calls and limited work hours that they knew about from the start of their residencies, factors also noted as important to women in other studies that examined how residency programs are selected [18]. Alternately, significantly more men than women rated as important residency programs that involved much "action" and required them to assume much responsibility for making clinical decisions. More men also considered it important that there be an opportunity to do research during residency. This greater importance placed by male students on being able to perform research was also found among the criteria for selecting a specialty and among the reasons for choosing medicine as a career. This observation might have implications for the future of Israeli academic medicine in light of the increasing proportion of women pursing a medical career. Despite the differences between the genders, over 80% of the male and female students answered that it was important/very important to select challenging and interesting residency programs that were characterized by good interpersonal relationships between staff members and that provided much teaching (Table 4). Furthermore, 71% of the students indicated that positive treatment by the institution was important when selecting a residency program (Table 5). This importance placed on the workplace atmosphere and didactics was also observed among students in other countries who were considering emergency medicine and obstetrics/gynecology residency programs, and rated a friendly environment as one of their most important selection criteria [19,20]. Similar results were reported from a survey of Canadian family practice residents, where more than 50% replied that the ability to learn and work in a friendly professional environment was an important factor in choosing a training program [21].

Previous studies of criteria students worldwide use to select a residency program showed that the program's reputation, prestige, and location are among the major factors students utilize when selecting a program [21-24]. These results were echoed by the responses of the Israeli students, where over 60% indicated that they wanted to attend a residency program in a specific location within Israel. Furthermore, over 50% of men and women students considered it important that the residency program be in a leading department in the specialty and located in a large university medical center, although excellence of the medical center was significantly more important to men than women when choosing a location for their residency. These results are in contradistinction to the almost absolute lack of interest among the medical students in doing residencies in hospitals located in the country's peripheral areas. This observation was especially interesting since the students in this study were attending medical schools located away from the major population center. However, only about 10% replied that the location of their medical school was an important factor in deciding the location of their residency program, and almost a third indicated that living in the major population center (the central part of the country) was an important factor. In fact the students' wishes to do their residencies in leading departments in their selected specialty located in large, excellent medical centers can be construed as a euphemism for the large university hospitals located in the major population center. These series of responses point to a major problem for the Israeli healthcare system with filling residency positions in hospitals in the periphery in the future. Recruiting medical students to residencies in peripheral (or rural) areas, and ultimately having them remain there to practice, is an issue that is discussed in investigations emanating from different parts of the world [25-27]. In many countries efforts have been made to increase the number of rural physicians. These efforts, which have met with varying degrees of success, include recruiting medical students who reside in rural areas, rural-oriented medical curriculum, and rural rotations during medical school and residency [25,27,28]. To increase the recruitment of quality medical students to residency programs in the country's peripheral areas, the Israeli healthcare leadership should consider similar initiatives. It is important to note that the newly signed collective bargaining agreement between the Israel Medical Association and the major employers provides for special incentives and additional salary for physicians working in the peripheral areas of the country.

This study also made additional important observations. Among them were the distinct differences in specialty and residency selection criteria patterns between students who expressed interest in family medicine, pediatrics, and internal medicine and those who did not. These different selection patterns could possibly be utilized to identify students as potential candidates to pursue primary care so that recruitment could be focused on them [29]. Another interesting observation was that only a little more than a third of the students rated as important a specialty that required much teamwork, while over half reported that the ability to work as an independent practitioner was an important criterion. With medicine becoming more complex and bureaucratic, physicians are less often solo practitioners and more often members of healthcare teams. In recognition of this trend, some medical schools have added inter-professional education to their curriculum [30].

The limitations of this study are its examining only a limited number of selection criteria, while other factors undoubtedly also play a part in the selection process. However, the factors included in the questionnaires were those deemed important in similar studies performed worldwide. The aim was to limit the questionnaire to two pages so that it would not appear daunting to these students, many of whom stated that it was the first time in their medical school career that they had been formally confronted with such questions. In fact, many remarked that the questionnaire would make them begin to think more about their selection of a career specialty. The study was also limited to students attending two of the four Israeli medical schools. This was an advantage since it permitted the investigators to focus on getting a high response rate, which improved the validity of the study. In Israel there are about 300 medical students per year so that this study of more than 200 students over two years represents over a third of the 600 fifth-year medical students. In addition, there were no significant differences between the data collected from the two medical schools. Another limitation was that the definition of a peripheral hospital was left to the student and was not a priori defined for them. Despite this lack of definition there were no differences in the responses between students in Jerusalem and Beer-Sheva. A further limitation is that this is an initial study that does not examine whether counseling or providing information during the fifth-year actually influences specialty choice. This should be the subject of a subsequent interventional study.

## Conclusions

The fifth-year Israeli medical students had already begun considering criteria to use when choosing a medical specialty and residency program. It is interesting to note that the criteria the Israeli students deemed important were similar to those considered important by students from other countries. This included wanting interesting, challenging, and advancing specialties with reasonable relationships between lifestyle and income. In addition, they did not want residency programs in the periphery (rural areas) of the country. The differences between the responses of male and female medical students were also comparable to the differences found among students in other countries. These findings are not unexpected in an increasingly globalized world and the characteristics of Generations X and Y. Furthermore, about eighty percent of the students had already identified one possible specialty as a career, which is also similar to the number observed elsewhere [4,5]. These observations, thus, identify the fifth-year of medical school as an appropriate time to provide students with information and guidance as they begin to solidify their perceptions and ideas about the various specialties. This observation is important given the lack of formal career counseling and mentoring provided to Israeli students by their medical schools and during internship (which are formally under the aegis of the medical schools). Studies such as the present one should thus serve as an impetus to medical educators and the leaders of the healthcare system to begin to take a greater interest in the career selection by medical students and interns [31]. Increased interest in specialty selection is especially timely since the Israeli healthcare system is bedeviled by a maldistribution of medical specialists, with shortages in key specialties, such as anesthesiology and general surgery, threatening the future functioning of the system [6,7]. Moreover, the leadership needs to use these data to design selection processes, medical school curriculum experiences, role model exposure, career counseling services, and incentives to address the shortages of physicians in certain specialties.

## Competing interests

The authors declare that they have no competing interests.

## Authors' contributions

CW - concept, design, data collection and analysis, and manuscript writing. RYZR - design, data collection and analysis, revision of manuscript. JES - concept, design, review and revision of manuscript. YGW - design, data analysis and interpretation, review of manuscript. AA - design, data collection, review and revision of manuscript. UE - design, review and revision of manuscript. HT - concept, design of questionnaire, data collection, review and revision of manuscript. All the authors read and approved the final manuscript.

## Authors' information

Charles Weissman is the Chairman of the Department of Anesthesiology and Critical Care Medicine, Hadassah - Hebrew University Medical Center and a Professor of Anesthesiology at the Hebrew University - Hadassah School of Medicine, Jerusalem, Israel.

Rachel Yaffa Zisk-Rony is research mentor for the pediatric and obstetrical nursing staff of the Hadassah - Hebrew University Medical Center and a faculty member at the Henrietta Szold School of Nursing of the Hebrew University and Hadassah, Jerusalem, Israel.

Josh Schroder is a resident in the Department of Orthopedic Surgery Hadassah - Hebrew University Medical Center and an Instructor of Orthopedic Surgery at the Hebrew University - Hadassah School of Medicine, Jerusalem, Israel.

Yoram Weiss is the Head of the Department of Anesthesiology, Division of Anesthesiology and Critical Care Medicine, Hadassah - Hebrew University Medical Center and an Associate Professor of Anesthesiology at the Hebrew University - Hadassah School of Medicine, Jerusalem, Israel. He is also an Adjunct Associate Professor in Anesthesiology at the University of Pennsylvania School of Medicine, Philadelphia, PA.

Alex Avidan is an attending anesthesiologist Department of Anesthesiology and Critical Care Medicine, Hadassah - Hebrew University Medical Center and a Lecturer in Anesthesiology at the Hebrew University - Hadassah School of Medicine, Jerusalem, Israel.

Uriel Elchalal is the Director of the High-Risk Obstetrics Clinic at the Hadassah - Hebrew University Medical Center and an Associate Professor of Obstetrics and Gynecology, Hebrew University - Hadassah School of Medicine, Jerusalem, Israel.

Howard Tandeter is the Director of the School of Continuing Education and a Senior Lecturer in Family Medicine at the Ben Gurion University School of Medicine, Be'er Sheva, Israel.

## References

[B1] Wright B Scott I Woloschuk W Brenneis F Career choice of new medical students at three Canadian universities: family medicine versus specialty medicine CMAJ 2004 170 1920 1924 10.1503/cmaj.1031111 15210640 PMC421719

[B2] Dikici MF Yaris F Topsever P Fillz TM Gurel FS Cubukcu M Gorpelloglu S Factors affecting choice of specialty among first-year medical students of four universities in different regions of Turkey Croat Med J 2008 49 415 420 10.3325/cmj.2008.3.415 18581621 PMC2443626

[B3] Scott IM Wright BJ Brenneis FR Gowans MC Whether or wither some specialties: a survey of Canadian medical student career interest BMC Medical Educ 2009 9 57 10.1186/1472-6920-9-57 PMC2749833 19732455

[B4] Compton MT Frank E Elon L Carrera J Changes in U.S. medical students' specialty interests over the course of medical school J Gen Intern Med 2008 23 1095 1100 10.1007/s11606-008-0579-z 18612751 PMC2517937

[B5] Babbott D Baldwin DC Jr Joll P Williams DJ The stability of early specialty preferences among US medical school graduates in 1983 JAMA 1988 259 1970 1975 10.1001/jama.1988.03720130034026 3346978

[B6] Nirel N Birkenfeld S Benbassat J Criteria for a medical specialty in crisis: a case study of general surgery and internal medicine Harefuah 2008 147 553 559 18693635

[B7] Weissman C Eidelman LA Pizov R Matot I Klein N Cohn R The Israeli anesthesiology workforce IMAJ 2006 8 255 260 16671362

[B8] Rabinowitz HK Diamond JJ Markham FW Santana AJ Increasing the supply of rural family physicians: recent outcomes from Jefferson Medical College's Physician Shortage Area Program (PSAP) Acad Med 2011 86 264 269 10.1097/ACM.0b013e31820469d6 21169776

[B9] Shye D Gender differences in Israeli physicians' career patterns, productivity and family structure Soc Sci Med 1991 32 1169 1181 10.1016/0277-9536(91)90094-S 2068600

[B10] Dorsey ER Jarjoura D Rutecki GW The influence of controllable lifestyle and sex on the specialty choices of graduating U.S. medical students, 1996-2003 Acad Med 2005 80 791 796 10.1097/00001888-200509000-00002 16123455

[B11] Furnham A Attitudes to the medical specialties: comparing pre-clinical students' perceptions of nine specialties Soc Sci Med 1986 23 587 594 10.1016/0277-9536(86)90152-8 3764508

[B12] Dorsey ER Jarioura D Rutecki GW Influence of controllable lifestyle on recent trends in specialty choice by US medical students JAMA 2003 290 1173 1178 10.1001/jama.290.9.1173 12952999

[B13] Skinner CA Re-inventing medical work and training: a view from generation X Med J Aust 2006 185 35 36 16813548 10.5694/j.1326-5377.2006.tb00449.x

[B14] Snafey HA Saalwachter-Schulman AR Nyhof-Young JM Eidelson B Mann BD Influences on medical student career choice: gender or generation? Arch Surg 2006 141 1086 1094 10.1001/archsurg.141.11.1086 17116801

[B15] Lefevre JH Roupret M Kerneis S Karila L Career choice of medical students: a national survey of 1780 students Med Educ 2010 44 603 612 10.1111/j.1365-2923.2010.03707.x 20604857

[B16] Jackson I Bobbin M Jordan M Baker K A survey of women urology residents regarding career choice and practice challenges J Womens Health 2009 18 1867 1872 10.1089/jwh.2008.1236 19951224

[B17] WINS White Paper Committee Benzil DL Abosch A Germano Im Glimer H Maraire JN Muraszko K Pannullo S Rosseau G Schwartz L Tudor R Ullman Zusman E J The future of neurosurgery: a white paper on the recruitment and retention of women in neurosurgery J Neursurg 2008 109 378 386 10.3171/JNS/2008/109/9/0378 18759565

[B18] Agaard EM Julia K Julien D Soloman I Tillisch J Perez-Stable EJ Factors affecting medical students' selection of an internal medicine residency program J Nat Med Assoc 2005 97 1264 1270 PMC2594785 16296217

[B19] DeSantis M Marco CA Emergency medicine residency selection: factors influencing candidate decisions Acad Emerg Med 2005 12 559 561 10.1111/j.1553-2712.2005.tb00899.x 15930408

[B20] Nuthalalpty FS Goepfert AR Jackson JR Owen J Do factors that are important during obstetrics and gynecology residency program selection differ by applicant gender? Am J Obstet Gynecol 2005 193 1540 1543 10.1016/j.ajog.2005.07.050 16202753

[B21] Lee J Alfieri M Patel T Lee L Choosing family medicine residency programs Can Fam Physician 2011 57 e113 e121 21520673 PMC3056704

[B22] Nuthalalpty FS Jackson JR Owen J The influence of quality-of-life, academic, and workplace factors on residency program selection Acad Med 2004 79 417 425 10.1097/00001888-200405000-00010 15107280

[B23] Davydow D Bienvenu OJ Lipsey J Swartz K Factors influencing the choice of a psychiatric residency program: a survey of applicants to the Johns Hopkins Residency Program in Psychiatry Acad Psych 2008 32 143 146 10.1176/appi.ap.32.2.143 18349335

[B24] Patel S Colaco HB Hossain FS Factors influencing foundation programme choice among medical students J Roy Soc Med (Special Report) 2010 1 1 4 10.1258/shorts.2009.100056 PMC2984331 21103096

[B25] Talley BE Moore SA Camargo CA Jr Rogers J Ginde AA Availability and potential effect of rural rotations in emergency medicine residency programs Acad Emerg Med 2011 18 297 300 10.1111/j.1553-2712.2010.00987.x 21401792

[B26] Henry JA Edwards BJ Crotty B Why do medical graduates choose rural careers? Rural Remote Health 2009 9 1083 1096 19257797

[B27] Curran V Rourke J The role of medical education in the recruitment and retention of rural physicians Med Teach 2004 26 265 272 10.1080/0142159042000192055 15203506

[B28] Pretorius RW Lichter MI Okazaki G Sellicki JA Where do they come from and where do they go: implications of geographic origins of medical students Acad Med 2010 85 S17 S20 20881695 10.1097/ACM.0b013e3181ed3e78

[B29] Tandeter H Granek-Catarivas M Choosing primary care? Influences of medical school curricula on career pathways IMAJ 2001 3 969 972 11794930

[B30] Blue AV Mitcham M Smith T Raymond J Greenbaum R Changing the future of health professions: embedding interprofessional education within an academic health center Acad Med 2010 85 1290 1295 10.1097/ACM.0b013e3181e53e07 20671454

[B31] Shah SU The medical students' dilemma: which postgraduate specialty to pursue? J Postgrad Med 2009 55 294 295 10.4103/0022-3859.58941 20083884

